# Influence of Landscape Structure and Human Modifications on Insect Biomass and Bat Foraging Activity in an Urban Landscape

**DOI:** 10.1371/journal.pone.0038800

**Published:** 2012-06-07

**Authors:** Caragh G. Threlfall, Bradley Law, Peter B. Banks

**Affiliations:** 1 Evolution and Ecology Research Centre, School of Biological, Earth and Environmental Sciences, University of New South Wales, Sydney, New South Wales, Australia; 2 Forest Science Centre, NSW Primary Industries, Beecroft, New South Wales, Australia; 3 School of Biological Sciences, University of Sydney, Sydney, New South Wales, Australia; University of Lancaster, United Kingdom

## Abstract

Urban landscapes are often located in biologically diverse, productive regions. As such, urbanization may have dramatic consequences for this diversity, largely due to changes in the structure and function of urban communities. We examined the influence of landscape productivity (indexed by geology), housing density and vegetation clearing on the spatial distribution of nocturnal insect biomass and the foraging activity of insectivorous bats in the urban landscape of Sydney, Australia. Nocturnal insect biomass (g) and bat foraging activity were sampled from 113 sites representing backyard, open space, bushland and riparian landscape elements, across urban, suburban and vegetated landscapes within 60 km of Sydney's Central Business District. We found that insect biomass was at least an order of magnitude greater within suburban landscapes in bushland and backyard elements located on the most fertile shale influenced geologies (both *p*<0.001) compared to nutrient poor sandstone landscapes. Similarly, the feeding activity of bats was greatest in bushland, and riparian elements within suburbs on fertile geologies (*p* = 0.039). Regression tree analysis indicated that the same three variables explained the major proportion of the variation in insect biomass and bat foraging activity. These were ambient temperature (positive), housing density (negative) and the percent of fertile shale geologies (positive) in the landscape; however variation in insect biomass did not directly explain bat foraging activity. We suggest that prey may be unavailable to bats in highly urbanized areas if these areas are avoided by many species, suggesting that reduced feeding activity may reflect under-use of urban habitats by bats. Restoration activities to improve ecological function and maintain the activity of a diversity of bat species should focus on maintaining and restoring bushland and riparian habitat, particularly in areas with fertile geology as these were key bat foraging habitats.

## Introduction

Urbanization radically alters land surfaces, habitat structure and ecological function well beyond the bounds of the city [1], [2]. Urban ecological studies typically focus on the patterns of abundance and diversity of species that remain in cities after such habitat loss. However, other more subtle mechanisms governing these patterns are now receiving greater attention, including competition, predation, altered temperatures and productivity (e.g [3], [4]).

Productivity is a concept that describes the flow of energy through ecosystems, mostly referred to as photosynthetic rate or net primary productivity (NPP) (e.g. [5]). Productivity is a key mechanism influencing diversity and abundance of plants and animals [6], [7]. Establishment of human settlements is influenced by factors including water availability, climate and soil fertility [8], typically causing them to coincide with areas of high productivity [9], [10]. Additionally, species richness of many (but not all) taxa increases with increasing NPP [9], as does abundance [11]. Hence, areas of high human population density were once typically biologically diverse, and some still are [12]. This global coincidence of humans and areas of high productivity poses a threat to biodiversity, urging the need to characterize the impact of increasing urbanization on diverse urban ecosystems, where studies have been limited to date.

Underlying geology, soil and foliage nutrients have been used as surrogate measures of productivity, as they play a role in shaping vertebrate distributions and abundance [13], [14]. Previous work shows increased levels of insect herbivory in sites with greater productivity, as indexed by variables including soil nitrogen and phosphorus, and foliar nitrogen [15]. Nitrogen is also a key limiting nutrient for herbivores [16], for example arboreal mammals, who show a preference for foliage of higher nitrogen content [17]. The question remains however, whether productivity continues to influence species abundance and distributions in cities despite enormous alterations to the ecosystem like vegetation clearing, increasing housing density and increasing ‘heat island’ effects (the increased temperatures experienced in cities due to increased impervious surface cover [1]), which may shift trophic relationships. These activities are likely to affect primary consumers, such as insects, as insects respond to increasing nutrients [18], temperature [19], and productivity [11], which could ultimately impact upon secondary consumers in higher trophic levels. Indeed, evidence to date suggests that insect densities can be greater in vegetated areas within cities, shown by higher total canopy arthropod abundances in urban remnants compared to non-urban sites [20], and in urban backyards with greater canopy cover [21].

Insectivorous bats are an example of a secondary consumer that may be impacted indirectly by urbanization, due to the interactive effects of landscape variables and increasing urbanization on insect densities. Indeed, bat activity has been previously shown to increase with increasing insect abundance in agricultural environments [22]. Recent investigations of bat activity in urban environments suggest that bats respond to soil nutrients, as bat activity and species richness is higher in areas on fertile soils [23], [24]. However, the actual mechanism supporting this increase has yet to be explored. Changes in nutrients through the output of waste water alters the composition and abundance of nocturnal flying insects and the presence or absence of certain microbat species [25]. Although overall bat activity is likely to be influenced by many factors, including roost availability, microclimate, habitat structure and energetic requirements [26], [27], [28], prey abundance and availability is also predicted to contribute significantly [26], especially to bat foraging activity [29], [30]. Investigations of total bat activity may reveal different results to investigations of feeding behaviour, as bat activity recorded using ultrasonic detectors (e.g. 23,24]) records all bat behaviours, including commuting, social activities, searching and foraging. One recent study showed that forest-town interface sites support greater feeding activity, however these sites do not have greater total bat activity [31], suggesting that the increases in insect resources at forest-town sites facilitates greater feeding activity only. Furthermore, Jung and Kalko [32] showed that feeding activity was lowest in city sites, despite no differences in insect abundance between sites, indicating that insects present in urban areas may be unavailable to bats, simply because some bat species do not inhabit these areas [23], [24], [31], [33], [34], [35], [36]. Hence, although it is expected that changes in urbanization, productivity and vegetation cover would affect bats in part via influencing the distribution and abundance of insects, a direct test focusing explicitly on bat feeding activity is needed to assess this.

We investigated whether underlying dominant geology (as a measure of soil nutrients), housing density and native vegetation cover influence the spatial distribution of nocturnal insect biomass (as a measure of prey productivity) and insectivorous bat foraging activity along the urban gradient in Sydney, New South Wales (NSW), Australia. Urban development has been non-random in this landscape, where the productive nutrient-rich plains have been preferentially cleared and developed, initially for agriculture then for housing; most native vegetation in the greater Sydney region remains on the steeper nutrient poor geology [37]. Sites of greater vegetation cover and structural complexity support greater insect abundance [21], [38], [39], [40], and as such, vegetation clearing in urban landscapes could negatively impact insects, and subsequently bats. Previous studies show that total bat activity, and the activity of certain species, including the common Gould's wattled bat *Chalinolobus gouldii*, and Eastern Bentwing bat *Miniopterus schreibersii oceanensis*, is influenced by landscape geology in the study area [23], [24], [36]. Our study explicitly examined this relationship in more detail, by investigating if increased insect biomass was a function of geology, and if this influenced bat feeding activity (rather than total activity). Difficulties exist in establishing direct relationships between predators and prey, because prey may be abundant but unavailable, for example insects in cluttered environments become unavailable as prey items to bats that cannot negotiate or efficiently echolocate in such habitat [41]. Hence, we aimed to investigate common explanatory variables of insect biomass and bat foraging activity, as a first step in teasing out the influence of productivity on primary and secondary consumers in urban landscapes. We hypothesize that dominant geology positively affects insect biomass via the influence of soil nutrients available for plant and thus insect growth, and that this pathway in turn positively influences the foraging activity of insectivorous bats. Specifically, we predict that landscapes with soils of higher nutrient content would support higher insect biomass, and greater bat foraging activity than landscapes with nutrient poor soils. We also predict that insect productivity and bat foraging activity is negatively correlated with increasing urbanization (housing density), and that productive landscapes with native vegetation cover support greater insect biomass, providing foraging habitat for bats.

## Methods

### Study area

**Figure 1:**
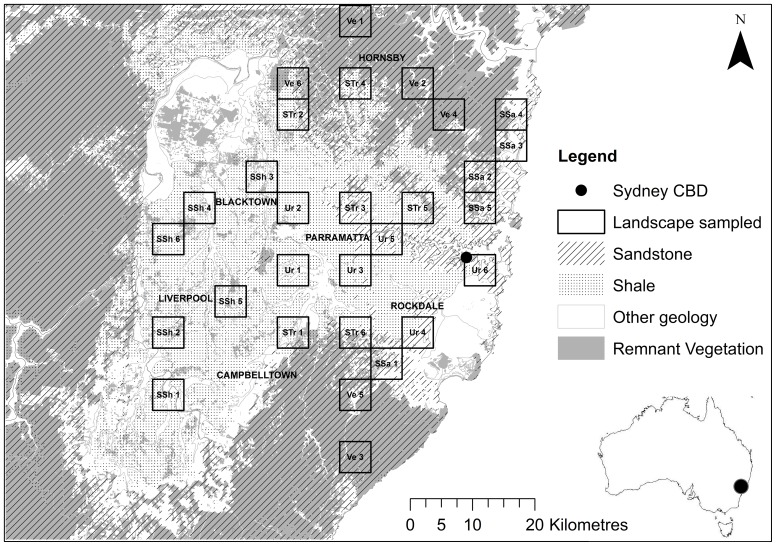
Map of sampled landscapes in Sydney, NSW, Australia. Landscapes include Urban (Ur, n = 6); Suburban Shale (SSh, n = 6); Suburban Sandstone (SSa, n = 5); Suburban Transition (STr, n = 6); and, Vegetated (Ve, n = 6) categories. Within each landscape, four elements were sampled: backyard, bushland remnant, riparian corridor and open space.

The study was carried out in a 4000 km^2^ area of the Sydney Metropolitan region, NSW, Australia. Sydney is Australia's oldest and largest city, founded in 1788 [37]. Sydney currently supports nearly 4 million people, and is rapidly expanding. There are two primary geologies of the area, the Wianamatta shale, including some of the Narrabeen group shales (hereafter shale) and Hawkesbury sandstone (hereafter sandstone) (Fig. 1). The soils on the shale plain are of higher fertility and nitrogen concentration [42], [43], and this area is the most highly developed and fragmented element of Sydney's landscape [37]. This contrasts to the vegetated sandstone plateaux, which contain most of Sydney's National Parks [37] (Fig. 1).

### Study design and landscape selection

Insects and bats were sampled in randomly selected 5×5 km ‘landscapes’ each within 60 km of Sydney's Central Business District (CBD) (following [24]) (Fig. 1). Landscapes were categorized based on the level of urbanization and remaining native remnant vegetation cover using Arc Map (ESRI, Redlands, California, USA, version 9.3) and GIS layers obtained from the New South Wales (NSW) Office of Environment and Heritage (OEH), NSW Department of Primary Industries (NSW DPI) and the Australian Bureau of Statistics (ABS). Landscapes were categorized as: urban (>5 dwellings/ha and <10% vegetation cover); suburban (2–5 dwellings/ha and 5–40% vegetation cover); and vegetated (<5 dwellings/ha and >40% vegetation cover). Within the ‘suburban’ landscape category, sites were further classified into ‘suburban shale’ (∼>80% of landscape dominated by shale), ‘suburban sandstone’ (∼>80% of landscape dominated by sandstone), and ‘suburban transition’ (∼>40% shale and ∼>40% sandstone transitional area). This classification was not possible in the ‘urban’ and ‘vegetated’ landscapes, as the underlying geology is predominately sandstone or shale, respectively. Four landscape elements were sampled within each landscape to investigate the contribution of different habitat types within the landscape units. These were: a) bushland (>2 ha mapped remnant vegetation); b) riparian areas (natural mapped waterway 2–10 m wide); c) open space (e.g. parkland); and, d) backyards. Bat and insect data were collected at 113 sites across the study region, within 29 defined landscapes (Fig. 1); six replicate landscape ‘blocks’ of each of the urban, suburban shale, suburban transition and vegetated categories, and five replicate ‘blocks’ of the suburban sandstone category. Three inaccessible sites were removed from the design that were from the ‘vegetated’ and ‘suburban transition’ categories. Any two elements were located greater than 500 m apart.

### Insect and bat sampling

Sampling occurred on mild nights during late spring-early summer (October – December) 2008, avoiding nights either side of the full moon, which is known to interrupt normal behaviours of insects and bats [44], [45]. Early summer coincides with the bat maternity period when resource requirements, especially for females, are likely to be highest [46]. This period is also when bat activity, as recorded via ultrasonic detection, is most reliable as a measure as it is very low during winter and artificially inflated after December, when the young of the year begin to fly [46]. All necessary permits were obtained for the described field studies, granted by permission from the OEH (Licence # S10860), and private land owners. During insect sampling, mean nightly temperature varied between 10–24°C, and averaged 16.8°C across all sampling nights. During bat sampling, mean nightly temperature varied between 7–23°C, and averaged 16.0°C; hence both data sets were collected during comparable weather. Data were collected on warm nights, and in the event of heavy rain or strong wind sites were re-sampled. Flying nocturnal insects were sampled within each element via the use of a black-light insect trap, using an 8-W fluorescent tube (Australian Entomological Supplies, Bangalow, Australia). The samples were taken at a random point within five metres of where bat activity was recorded (see below), and the trap was deployed at ground height for one entire night. Samples were taken on an alternate night to bat data collection to avoid any disturbance to the normal flight behaviours of bat species in response to the presence of black light, as certain species may be absent from well-lit sites, including species with low intensity calls [47]. Although not ideal, the two samples were taken within the same season, typically within two weeks of each other and in comparable weather, and as such are considered a reasonable representation of insect biomass and bat activity of each site. Light traps were activated for the same period as bat sampling (1800–0630 h), which encompasses the period from before and after sunset/rise. Timers connected to the traps controlled their activation. Insect samples were stored in 70% ethanol until identification. Individuals were sorted into three categories (moths, beetles and others, according to [48]), counted and then oven dried at 60°C until a constant mass was achieved, usually 4 days. Mass was recorded to the nearest 0.001 g. Moths and beetles were separated as they are major prey items for many bat species in our study area [22], [46]; remaining insects were classed as ‘other’. Dry mass of a known number of individuals was estimated from subsamples. Regression equations were developed to predict the relationship between number of individuals and the total dry mass per category (r = 0.7–0.95). These regression equations were then used to predict the dry mass of insect samples.

Bat foraging activity was sampled using Anabat detectors (Titley Electronics, Ballina, Australia). Each of the four elements within a landscape was sampled remotely for two full consecutive nights from sunset to sunrise (1800–0630 h). The microphone was set at 1 m above the ground at an angle of 45° and detectors were placed on flyways, or facing gaps in vegetation. Sampling along flyways has been shown to maximize species detection using ultrasonic recordings [49], and whilst this method may be biased towards species with loud calls, we have avoided making species comparisons to reduce the effect of this. The placement of light traps also minimized between site variations, as all were placed in an open area, flyway, or an area with minimal vegetation coverage. Bat passes were recorded onto a CF storage card via a zero-crossing interface (Z-CAIM, Titley Electronics). Bat passes, defined here as a pass with three or more pulses, were stored as a single file and processed by Anascheme software [50]. Anascheme uses regional identification keys to identify passes to taxa by extracting a range of call parameters [50]. We used an existing identification key developed to identify the species in this region [50].

Foraging calls (feeding buzzes) within bat passes were distinguished from normal search phase calls for the purposes of this study, using a filter in Anascheme (B. Law unpubl. data), which recognised short sequences of steep linear calls produced in rapid repetition, typical of feeding buzzes [51]. In a sample of 90 manually identified feeding buzzes our filter recognised 74%. Testing on non-feeding buzzes revealed that linear *Nyctophilus* calls (n = 46) were not identified as feeding buzzes, but occasional clutter calls from species calling at high frequencies were confused with feeding buzzes. Accordingly, all files matching our feeding buzz filter were manually checked to exclude non-feeding buzzes. This process allocates feeding buzzes to passes that are identified to species, and to those for which species identification was not assigned. Foraging activity was expressed as the number of bat passes containing a feeding buzz, where feeding from all species recorded was combined to assess overall bat foraging activity. The number of calls containing a feeding buzz as a proportion of total bat activity was also calculated.

Ambient temperature was measured every 15 min using temperature i-button data loggers (Maxim, Sunnyvale, Canada) for the period the detectors and light traps were operating (1800–0630 h). Maximum and average nightly temperature over the survey nights were calculated for each element. For elements where data were missing (n = 7), it was supplemented with hourly measurements from the nearest weather station [52].

### Environmental variables

We established two vegetation sampling transects to describe the vegetation structure within each element. These were 50 m long, and measurements were taken at five random points along each transect. Vegetation clutter affects bat mobility and prey detection [53] and was quantified by measuring projective foliage cover and strata height. Foliage cover was visually estimated for the ground strata, understorey and canopy at each point and was categorized as 1 (<10% cover), 2 (10–29%), 3 (30–49%), 4 (50–69%) and 5 (*>*70%) [53]. The height of each stratum at each point was measured using a clinometer or tape measure. This height was then multiplied by its cover score to give a weighted cover score for each stratum. These were then added together to give an average vegetation clutter score (range 0.25–114). The number of visible lights (street lights, building lights within 100 m) surrounding each detector/light trap site were also counted.

Landscape variables were calculated for each element using Arc Map. The distance (km) to the nearest native bushland (>0.5 ha) and mapped watercourse were measured using 1∶100 000 scale GIS mapping of drainage and vegetation extent. The amount of native bushland (ha) and housing density (houses/ha) within 500 m, 3 km and 5 km of each element was calculated. The total percent of sandstone based geology and shale based geology were also calculated for each landscape, using the 1∶250 000 GIS mapping of the Geological Map Sheet for Sydney (NSW DPI). Landscape heterogeneity (number of land cover types) per landscape was also recorded, using the ABS 2006 Census data, following Threlfall *et al.* [24].

### Statistical Analysis

Analyses were carried out using JMP (SAS Institute, version 7.0), unless otherwise stated. Our sampling strategy did not influence the results as calendar date was not correlated with insect biomass or bat feeding activity (Spearman's rank correlation r<0.1, p≥0.2), however, we acknowledge bat foraging activity and insect biomass are likely to change throughout the year. To assess spatial autocorrelation we calculated Moran's I for total insect biomass and bat foraging activity, in Arc Map.

Initially, we assessed variation in insect biomass and bat foraging activity across our landscape categories. A two-factor Analysis of Covariance (ANCOVA) was used to assess differences in total insect, moth, beetle and other biomass, between landscape categories and landscape elements. The factors tested were landscape category (n = 5), landscape element (n = 4), and nightly average temperature (°C) as the covariate. ‘Landscape block’ was added as a random term nested within landscape category, to account for replication at the landscape element level being shared at the landscape category level. Insect biomass was log x+0.01 transformed, so data conformed to the assumptions of normality and homogeneous variances. Five sites were removed from the moth, beetle and ‘other’ data sets, as samples had degraded and could not be completely sorted to Order. Specific planned contrasts were conducted (rather than all pair-wise comparisons) to test whether suburban shale landscapes differed to suburban sandstone and transition across all elements, and secondly whether vegetated landscapes differed to suburban landscapes (combined) or urban landscapes, across all elements. Using these contrasts, we did not test for example, if suburban sandstone differed from transition, only if these two landscapes differed from suburban shale, as the latter was predicted to have the highest insect biomass. We employed an α-level of 0.05. Bat foraging activity data could not be transformed to meet the assumptions of normality and homogeneous variances, and as such, a conservative approach was taken and these data were assessed via a Chi-squared Goodness of fit test that compared the observed number of feeding buzzes per landscape category and element to that expected (equally distributed). Landscape element within landscape category comparisons of feeding activity were not conducted due to violations of the assumptions of chi-squared tests.

Secondly, we assessed if the measured environmental variables explained the variation in insect biomass and bat foraging activity. Linear relationships between insect biomass, bat foraging activity and site and landscape characteristics were weak (Spearman r≤0.2, *P*>0.1), so instead a Classification and Regression Tree (CART) was used to identify threshold responses. This method uses a recursive data partitioning algorithm to initially split the data based on a single best predictor variable, one which minimises the variance in the response, resulting in two mutually exclusive groups [54]. This process is then repeated for subsequent groups. The output of this method is a tree with various branches and terminal nodes, where the splits represent a simple rule [54]. The most parsimonious model was refined via a cross validation procedure. The number of nodes and deviance explained by additional nodes was assessed via the cost-complexity parameter k. Optimal tree size was determined via the change in deviance explained with increasing tree size, and increasing k. Performance of the regression tree was assessed via a correlation of observed and expected values, and the R^2^. We constructed separate trees for insect biomass and bat foraging activity. We firstly constructed models using insect biomass as a response variable (total insects, and then moths and beetles separately). We then constructed a model using bat foraging activity as a response variable (both the number of passes containing feeding buzzes, and the proportion of feeding activity), where insect biomass variables (including the biomass of moths, beetles and ‘others’) were added as predictors to the bat foraging model. This foraging model was constructed to investigate whether insect biomass explained variations in bat foraging activity, in addition to the influence of the environmental variables. Insect biomass was significantly auto-correlated (Moran's I = 0.37, *P*<0.05), hence site locations (x, y co-ordinates) were added to the predictor variables for this analysis. Foraging activity was not significantly auto-correlated (Moran's I = 0.04, *P*>0.05). Response variables were log x+0.01 transformed to improve model performance. Analyses were conducted using the ‘tree’ package [55] in R [56].

## Results

More than 60 000 nocturnal flying insects were collected using the light traps. Average insect biomass (dry mass) per site was 1.48±0.30 g, and ranged between <0.001 g–17 g. Three sites yielded no insects (two sandstone backyards and one transition bushland site), where no obvious trap failure occurred. Of the total biomass collected, 39.8% of the mass was accounted for by Coleoptera (beetles), and 15.7% by Lepidoptera (moths). The remainder comprised mainly of Diptera (flies), Hemiptera (bugs), Hymenoptera (wasps, bees and ants) and Isoptera (termites), which were classified as ‘other’ during sorting. Anabat detectors recorded 7767 bat passes from 17 taxa. The average nightly activity was 34.5±4.2 passes/night, where three species contributed the most to this activity: Gould's wattled bat *Chalinolobus gouldii*, the eastern freetail bat *Mormopterus* sp.2 [57], and the little forest bat *Vespadelus vulturnus*.

### Insect biomass along the urban gradient

**Figure 2:**
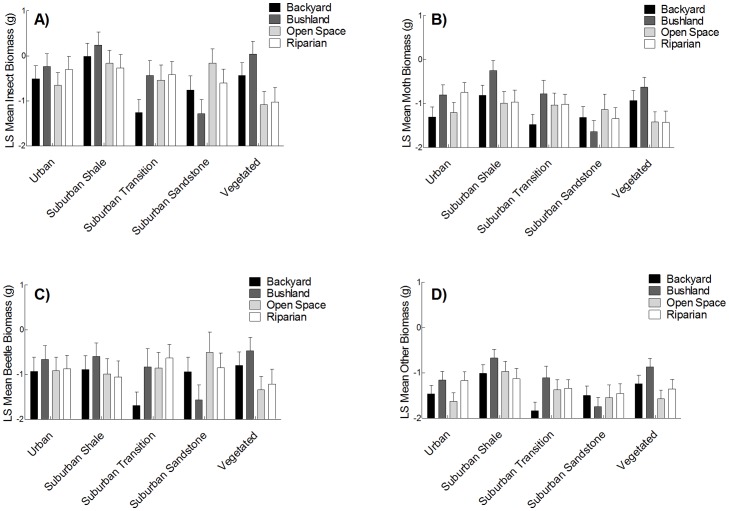
Nocturnal insect biomass (g) for each landscape element across landscape categories. (A) total biomass; (B) moth biomass; (C) beetle biomass; and (D) other biomass. The data are log (x+0.01 transformed) Least Squares means (± standard error), after adjusting for average nightly temperature. Results of planned contrasts (which combine categories) are included in the text.

The ANCOVA of total insect biomass revealed a significant landscape category by element interaction (Table S1, Fig. 2A). Insect biomass also significantly increased with increasing nightly temperature (Table S1), and varied among the replicate landscape blocks (Table S1). *A priori* contrasts to explore the interaction term revealed that within the suburban landscapes, shale backyards had insect biomass 36 times greater than transition backyards (t_2, 68_ = 3.1, *P* = 0.003), but did not differ compared to sandstone backyards (t_2, 68_ = 1.76, *P* = 0.08). Shale bushland also had insect biomass which was two orders of magnitude greater than sandstone bushland (t_2, 68_ = 3.54, *P* = 0.0007), but did not differ compared to transition bushland (t_2, 68_ = 1.53, *P* = 0.13). Open space and riparian elements did not differ significantly between the suburban landscapes (all *P*-values>0.05). Open space elements within vegetated landscapes had significantly lower insect biomass than within suburban landscapes (combined across all geologies) (t_2, 68_ = −2.33, *P* = 0.02), but not urban landscapes (t_2, 68_ = −1.06, *P* = 0.29). Insect biomass in riparian elements within vegetated landscapes was not significantly different to urban landscapes (t_2, 68_ = 1.70, *P* = 0.09), or suburban landscapes (t_2, 68_ = 1.63, *P* = 0.11). Backyard and bushland elements did not differ significantly between vegetated and suburban, or vegetated and urban landscapes (all *P*-values>0.05, Fig. 2A). Moth, beetle and ‘other’ biomass did not significantly vary between landscape categories or elements (Table S1, Fig. 2B–D). However, moth biomass varied among replicate blocks, as did beetle and the ‘others’ biomass, along with increasing temperature (Table S1).

### Bat foraging activity along the urban gradient

**Figure 3:**
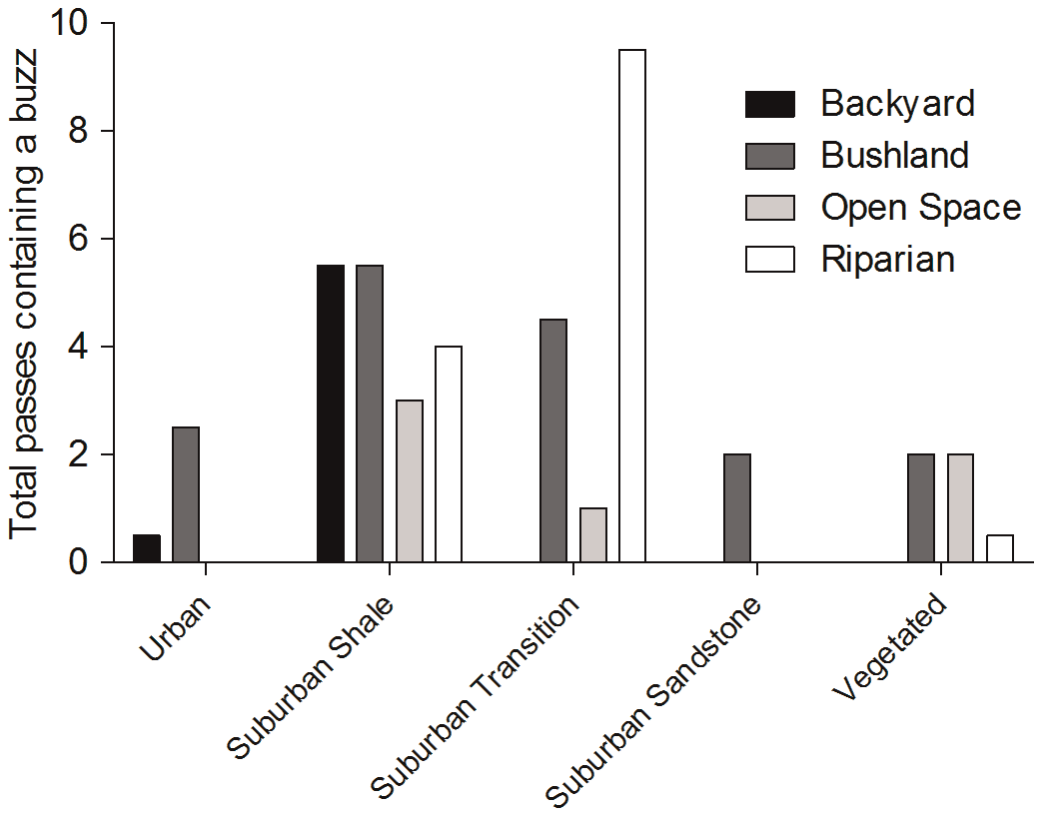
Total bat passes containing a feeding buzz. Recorded in each of the landscape categories and landscape elements (Note: analysis was done separately on the categories and elements due to the number of zeros recorded).

Eighty-five feeding buzzes were recorded in total (1.09% of total passes) at 25% of sites. Feeding buzzes were recorded mainly from *Chalinolobus gouldii* (28%) and *Vespadelus vulturnus* (18%). The observed frequency of feeding buzzes differed between landscape categories compared to expected (χ ^2^ = 26, d.f. = 4, *P*<0.001). There was more feeding activity in suburban shale and transition landscapes, and less feeding than expected in urban, vegetated and suburban sandstone landscapes (Fig. 3). These data are consistent with the finding that there was greater insect biomass in elements within the suburban shale landscapes, and less biomass in elements of the vegetated landscapes. The observed frequency of feeding buzzes differed between landscape elements (χ^2^ = 8.3, d.f. = 3, *P* = 0.039), with more feeding buzzes recorded in bushland and riparian elements and less than expected in backyards and open space (Fig. 3). Feeding activity within each element was biased towards suburbs with fertile soils, with 96% of feeding buzzes in riparian elements, and 60% of feeding buzzes in bushland elements occurring within suburban shale and transition landscapes. Additionally 92% of feeding in backyard elements and 50% in open space elements occurred in suburban shale. Shale suburbs recorded up to six identifiable taxa foraging, including *C. gouldii*, *C. morio*, *Mormopterus norfolkensis*, *Miniopterus schreibersii ocenaensis*, *Tadarida australis* and *V. vulturnus*. Transition suburbs recorded three of these taxa, in addition to *Scotorepens orion*. Sandstone suburbs recorded feeding activity by *V. vulturnus* in addition to the cave-dwelling *C. dwyeri*. *Chalinolobus gouldii* was the only species recorded feeding in the urban and vegetated landscapes, however several buzzes could not be identified to species.

### Predictors of insect biomass and bat foraging activity

**Figure 4:**
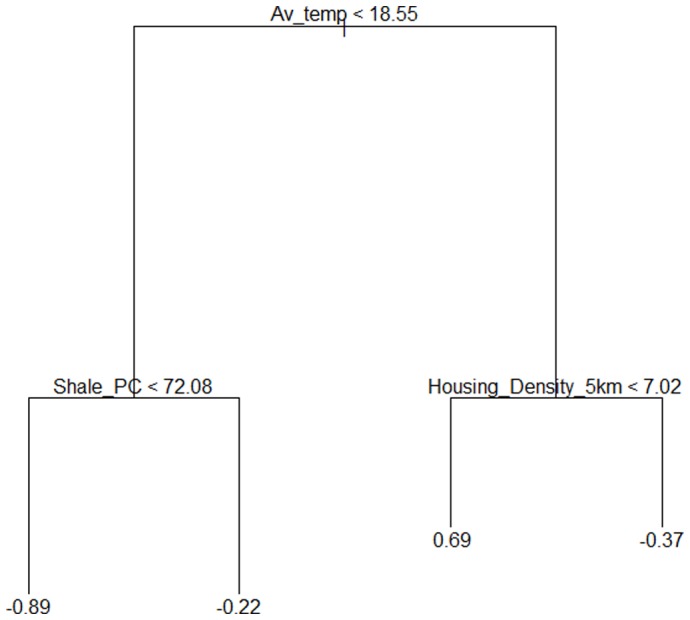
Regression tree for total insect biomass. Each split corresponds to a rule which is displayed with the variable causing the split (Condition<x, untransformed data). To investigate each condition proceed to the left or right branch of the node, following the less than or greater than signs. Values at the base of each node (vertical lines) represent mean insect biomass (log x+0.01) for that condition. Av_temp = average nightly temperature (°C) for each site during the sampling period; Shale_PC = the percentage of shale geology in each landscape sampled; Housing_Density = number of houses/ha measured within 500 m, 3 km and 5 km radii of each site.

Using regression tree analysis, we examined whether measured environmental variables (see Methods: Environmental variables) explained variation in insect biomass and bat foraging activity. Using this technique, three variables were identified as good predictors of insect biomass (Fig. 4). These three variables were also the most important predictors in regression trees for moth and beetle biomass (graphs not shown). The condition that led to the highest total insect biomass occurred in sites where the average nightly temperature was 18.5°C or above, with a housing density of 7 houses/ha or less, within a 5 km radius (Fig. 4). The condition that led to the lowest biomass occurred in sites where the average nightly temperature was below 18.5°C and less than 72% shale in the landscape occurred. All other variables were omitted from the final model. The residual mean deviance of the final insect biomass model was 0.51, with an R^2^ of 0.71.

**Figure 5:**
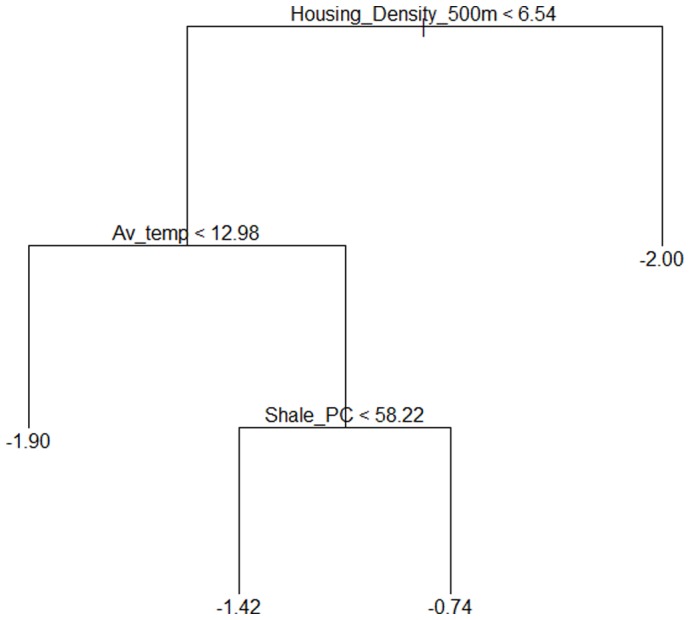
Regression tree for foraging activity. Each split corresponds to a rule which is displayed with the variable causing the split (Condition<x, untransformed data). To investigate each condition proceed to the left or right branch of the node, following the less than or greater than signs. Values at the base of each node (vertical lines) represent mean number of passes containing a feeding buzz (log x+0.01) for that condition. Variables follow those defined in Figure 4.

Using regression tree analysis, the same three variables were identified as good predictors of bat foraging activity, namely average nightly temperature, housing density and % shale in the landscape (Fig. 5). These variables were also the most important predictors in a regression tree of the proportion of foraging activity (graph not shown). However, unexpectedly there was no direct relationship between insect biomass and bat foraging activity, and consequently insect variables were not included in the final model. The condition that led to the highest foraging activity occurred in sites with a housing density of 6.5 houses/ha or less within a 500 m radius, average nightly temperature of 13°C or above and greater than 58% shale in the landscape (Fig. 5). The condition that led to the lowest foraging activity occurred in sites with a housing density greater than 6.5 houses/ha within a 500 m radius. The residual mean deviance of the final foraging activity model was 0.61, with an R^2^ of 0.54.

## Discussion

Urbanization has the potential to significantly alter ecological interactions, and we found that it plays a role in shaping spatial patterns of nocturnal insect biomass and the feeding activity of microbats. Nocturnal insect biomass and bat foraging activity varied between landscape categories based on geology and human modifications, including the loss of native vegetation cover and increased housing density. This is consistent with studies suggesting that insects, particularly moths, are in decline likely due to land use modification like urbanization [58], which in turn may cause a decline in bat foraging activity in urban areas [29], [31]. The most common bat species in the study area were *C. gouldii*, *Mormopterus* sp.2 and *V. vulturnus*, which primarily prey on moths, beetles and bugs [46]. However, insect variables were not directly responsible for explaining variation in bat foraging activity. One explanation for this is that prey may be unavailable to bats in highly urbanized areas if these areas are avoided by many species, suggesting that reduced feeding activity may reflect under-use of urban habitats by bats (discussed in more detail below).

### Changes in insect biomass

Insect biomass of bushland and backyard elements was greater in suburban landscapes located on fertile geologies, and this coincided with greater feeding activity of insectivorous bats. It is unclear why suburban transition backyards had lower insect biomass compared to shale, but not compared to sandstone backyards, however we did not record any details about how backyard gardens were managed (e.g. amount of top soil added, level of garden watering, etc), which potentially varies with socio-economic background, and could contribute to changes in invertebrate abundance and diversity [21], [59]. As predicted, however, bushland elements in suburban shale landscapes had significantly higher insect biomass compared to bushland elements in sandstone landscapes. Soils produced on shale geologies in the region are of higher fertility and nitrogen concentration than the soils derived from sandstone [42], [43]. Soil nitrogen and phosphorus measured in a mainly shale remnant in the area had almost double the concentrations recorded for undisturbed sandstone remnants [60], [61]. In addition, bushland on productive soils in southern NSW support flying insect densities more than an order of magnitude greater than bushland on Sydney sandstone [62], [63], likely due to the increased nitrogen in plant tissues, which increases abundances of herbivorous insects [18]. Geology has been shown to influence the distribution and abundance of various arboreal marsupials in forested regions in Australia, through differences in foliage nutrient concentration [13]. As such, it is likely that increased soil nutrients in fertile bushland remnants play a role in supporting greater diversity and activity of bats [24] due to increases in prey abundance. Indeed, it is possible that insect-bat relationships may be moderated by geology in urban landscapes like Sydney, with both nutrient rich and poor soils, which in turn influence habitat quality.

Contrary to our predictions, we found little direct influence of greater native vegetation cover on insect biomass or bat foraging activity. Previous work suggests insects are more abundant and diverse in areas of greater structural complexity and canopy cover, due to a greater diversity of habitat niches and increased food resources [38], [39], [40]. As such, we expected that the naturally vegetated landscapes in our study would have high insect biomass and provide foraging grounds for bats. Instead, suburban and urban landscapes had insect biomass equal to vegetated landscapes, yet they are the most cleared landscapes in the region, and higher insect biomass in fertile suburban landscapes was consistent with increased foraging activity of bats. However, our regression tree analysis showed that both insect biomass and bat foraging activity was lowest in areas with high density housing (which are often areas with the least vegetation cover), indicating that within the ‘urban’ landscape category variation in housing density influenced both insects and bats. Hence, although no direct relationship with vegetation cover was found, this may simply reflect the variable's negative correlation with housing density (Pearson's r = −0.6), as well as the greater artificial lighting levels in these areas. The negative relationship of insect biomass with housing density was expected, as urban centres are increasingly covered by impermeable surfaces such that net primary productivity is often lower than it would have been prior to the onset of urbanization [64]. Additionally, increasing human population density can be negatively associated with invertebrate diversity in backyards [59]. However, this effect may be counter-balanced by increased watering and nutrients in backyards, which is possibly the reason behind equal insect biomass in backyards in urban, suburban and vegetated landscapes. Additionally, no structural vegetation characteristics, including vegetation clutter (a measure similar to structural complexity), were important in explaining the variation in insect biomass, or bat foraging activity. Hence, our results suggest housing density is a better predictor than vegetation attributes of decreasing insect biomass and bat foraging behaviour in Sydney's landscape.

### Implications for bat foraging

Bat foraging activity was highest in fertile landscapes, suggesting that higher prey biomass (in the shale sites only) supports greater foraging resources for bats, a result similar to that found in agricultural landscapes [65]. In particular, we found that bushland and riparian elements within fertile suburban landscapes were frequent foraging grounds for a variety of species, demonstrating the conservation value of these habitats. Most riparian sites sampled had at least some riparian vegetation, which may explain why insect biomass did not differ between any riparian element sampled. Greater feeding activity here could reflect greater bat species richness, or greater roosting opportunities [36]. However, in other parts of the landscape, including urban and vegetated landscapes, insects may be unavailable to some bat species due to morphological constraints on prey detection [66] and flight [28]. Additionally, recent investigations of bat-insect relationships suggest that bat activity is constrained by prey accessibility, rather than just prey abundance *per se* [41], [67]. It is interesting to note that *C. gouldii* was the only species recorded feeding in the urban category. This species is from the edge – open adapted guild, with fast, agile flight and a low frequency modulated echolocation call, typically around 30 kHz [46], [67]. The ecomorphology of this species may give it an advantage over others [24], allowing it to successfully forage in relatively open sites where other species considered sensitive to urbanisation, including slow flying species that glean, or species with higher frequency calls (which are attenuated in open areas) [24], [36], may be less efficient [28]. Indeed, we found species with these traits foraging in the suburban shale and transition landscapes, perhaps because of the combination of increased insect resources and tree cover. Additionally, *Miniopterus schreibersii oceanensis* may have foraged in urban areas, but feeding buzzes were not detected. This is because their feeding buzzes are not distinctive on Anabats with a division ratio of 16 [68]. Given the relatively high activity of this edge-adapted species in urban areas [24], [69], we assume it also feeds there, and this species was recorded feeding in the suburban shale landscapes.

Prey biomass alone did not explain greater bat feeding activity, as the regression tree analysis did not include prey biomass in the final foraging model. Direct correlations between predator activity and prey abundance can be hard to establish (e.g. [25], [70]), particularly when preferred prey are only a subset of what is sampled, and prey abundance may not indicate prey availability, as can be the case for certain bats [31], [41]. Greater feeding activity may be associated with sites that harbour a different suite of prey for bats, as community composition of arthropods has been shown to vary between remnant type and between urban land uses [20], [71]. Although moths and beetles did not differ in total biomass across the study area, the composition of species within these categories, in addition to the composition of the ‘other’ category could also influence bat feeding activity, especially for smaller, less common species. While the diet of most bat species in our study area is opportunistic [72], some bats have a specialised diet with their activity being correlated with preferred prey [73]. Some of the bat species in Sydney have more specialised diets [46], however the diet of many species is unknown [46]. Additionally, aside from restrictions of prey accessibility, other factors specific to urban areas could influence overall bat activity and foraging activity, including roost availability [74], proximity to bushland [75], negative species interactions [23], or avoidance of features of urban habitats like increased road density [76] and artificial light [77], restricting how many bats occupy any given area. Hence, variations in foraging activity unrelated to prey biomass indicates that some urban areas do not support feeding activity, possibly due to other factors that reduce the presence of bats in general, such as a lack of roost sites.

### Management Implications

We suggest that management and restoration activities in urban and suburban areas focus on habitats highlighted as important feeding grounds, especially bushland and riparian areas on productive geologies. Although prey items declined in areas of high housing density, we still recorded one common species of bat (*C. gouldii*) feeding in these areas. Urban bushland remnants are often overgrown with weeds and it is possible that restoration activities in these areas could benefit bats. The one urban restoration study undertaken to date showed that prescribed burning and the removal of invasive weed species promoted general bat activity [78], however how such actions influence prey availability and bat foraging activity is unknown. The retention of bushland should be a priority, particularly in fertile suburbs, as these areas contained insect biomass two orders of magnitude greater than less fertile suburbs. Additionally, restoring riparian habitats would be beneficial for both insects and bats. Improving canopy cover, for example in private backyards and public managed open spaces, could be beneficial to bats as they can forage around scattered and isolated trees [22], with three-fold increases in bat richness associated with the presence of 3–5 trees per 2 ha in agricultural areas [79]. However, whether increases in scattered tree cover in urban areas would facilitate greater bat foraging activity has yet to be demonstrated. It should also be acknowledged that the extensive areas of National Parks that surround Sydney on sandstone geology support lower insect biomass compared to sites with more shale in the landscape, and as a result fewer foraging opportunities for a variety of insectivorous bat species. It is therefore necessary for urban planners to appreciate the mechanisms influencing trophic structure in cities as demonstrated here, in order to facilitate continued ecological functions across urban landscapes. We acknowledge our study is of just one city, and encourage testing of our hypotheses in other cities.

## Supporting Information

Table S1 Two–way analysis of covariance of insect biomass.
(DOC)
