# Supplementary material for: Influence of Landscape Structure and Human Modifications on Insect Biomass and Bat Foraging Activity in an Urban Landscape
Source: PLoS One. 2012 Jun 7;7(6):e38800. doi: 10.1371/journal.pone.0038800 (PMC3369849; doi:10.1371/journal.pone.0038800)
Supplement: Table S1 — Two–way analysis of covariance of insect biomass. (DOC) [file pone.0038800.s001.doc]

**Table S1:** **Two–way analysis of covariance of insect biomass.**

| **Variable** | **d.f.** | ***F*** | ***P*** |
| --- | --- | --- | --- |
| 1. **Total insect biomass** |  |  |  |
| Landscape | 4, 24 | 1.87 | 0.15 |
| Element | 3, 68 | 0.69 | 0.56 |
| Landscape x Element | 12, 68 | 1.89 | **0.05** |
| Temperature | 1, 68 | 7.05 | **0.01** |
| Block (Landscape) | 24, 68 | 1.85 | **0.03** |
| 1. **Total moth biomass** |  |  |  |
| Landscape | 4, 26 | 1.37 | 0.27 |
| Element | 3, 63 | 2.18 | 0.10 |
| Landscape x Element | 12, 63 | 1.40 | 0.19 |
| Temperature | 1, 63 | 1.52 | 0.22 |
| Block (Landscape) | 24, 63 | 2.05 | **0.01** |
| 1. **Total beetle biomass** |  |  |  |
| Landscape | 4, 26 | 0.09 | 0.99 |
| Element | 3, 63 | 0.41 | 0.75 |
| Landscape x Element | 12, 63 | 1.38 | 0.20 |
| Temperature | 1, 63 | 8.91 | **0.004** |
| Block (Landscape) | 24, 63 | 1.84 | **0.03** |
| 1. **Total other biomass** |  |  |  |
| Landscape | 4, 26 | 1.87 | 0.15 |
| Element | 3, 63 | 2.46 | 0.07 |
| Landscape x Element | 12, 63 | 1.15 | 0.34 |
| Temperature | 1, 63 | 8.5 | **0.005** |
| Block (Landscape) | 24, 63 | 2.63 | **0.001** |

a) total insect biomass; b) total moth biomass; c) total beetle biomass; and d) total other biomass, in relation to landscape category, landscape element and average nightly temperature (ºC). D.f. are degrees of freedom. Insect data are log (x +0.01) transformed. Bold values indicate significant differences (α ≤ 0.05).
